# Editorial: Insights in Experimental Pharmacology and Drug Discovery: 2021

**DOI:** 10.3389/fphar.2022.870830

**Published:** 2022-02-28

**Authors:** Andres Trostchansky, Salvatore Salomone

**Affiliations:** ^1^ Facultad de Medicina, Universidad de la República, Montevideo, Uruguay; ^2^ Department of Biomedical and Biotechnological Sciences, University of Catania, Catania, Italy

**Keywords:** experimental pharmacology, drug discovery, natural compounds, pain—drug therapy, Alzhaimer’s disease (AD)

This Research Topic was intended to focus on new insights, novel developments, current challenges, latest discoveries, recent advances, and future perspectives in Experimental Pharmacology and Drug Discovery. When launching this section, 12 years ago, we stated that it would deal with the analysis of those biological mechanisms targetable by drugs and/or affecting their action (Salomone, 2010). This analysis may be carried out at molecular, cellular, organ/organism level, with a further fourth level of analysis, individualized pharmacology, which takes into account the impact of genetics and epigenetics on drug efficacy and toxicity, to optimize drug therapy for each individual. Drug discovery may move from new chemical entities, conceived to target specific molecular and cellular processes relevant for a disease mechanism, but often takes the reverse way, which is moving from approved drugs to the study of their mechanisms, potentially exploitable to cure other, often unrelated, diseases (drug repurposing). Unexpectedly, our section has also seen a continuously growing number of submissions focusing on natural compounds, which are part of the traditional pharmacopeia in some cultures, particularly the Chinese one, but whose specific mechanisms are not known. These studies, when carried out according to the standards of modern pharmacology (pure substances, precisely known concentrations/doses, concentration-response relationship), may provide useful information not only to understand and implement the correct use of traditional medicine but, more importantly, to provide new lead compounds, specifically directed toward relevant pathophysiological targets.

Five original papers of this RT focus on natural compounds. Wei et al. report on apigenin, a hydroxy flavone active component of some herbal extracts, proposed to treat xerostomia. Based on the prevalence of xerostomia in menopausal women, they hypothesized that sex steroids may be involved in this condition, a mechanism also related to aquaporin 5 expression. Data obtained *in vitro* (human salivary gland cells challenged with estradiol) and *in vivo* (ovariectomized mice) indicated that apigenin upregulates aquaporin 5. This finding was paralleled by functional improvement *in vivo*, e.g., restoration of saliva flow rates by apigenin or estradiol, presumably through ERα signaling. Thus, apigenin appears as a potential therapeutic approach to treat xerostomia, particularly when associated with estrogen reduction. Further studies may indicate additional therapeutic uses of apigenin in conditions where ovary endocrine function is reduced. Qian et al. report on the effects of β-sitosterol, a plant-derived sterol, on synovial angiogenesis, as a potential treatment for rheumatoid arthritis. By using *in vitro* (human umbilical vein endothelial cells) and *in vivo* (collagen-induced arthritis mice) models, the authors tested the hypothesis that sitosterol inhibits VEGF signaling. *In vitro* data showed that β-sitosterol decreased cell proliferation and migration, while *in vivo* data showed a decrease of the swelling degree and the damage of bone and cartilage, inhibition of the synovial angiogenesis, and reduced expression of VEGFR2. These data, while providing a mechanistic rationale for β-sitosterol use in chronic articular inflammation, by showing anti-VEGF effects may suggest further investigations for its effects in experimental disease models involving VEGF signaling, and/or lead optimization to increase potency, specificity, and selectivity. He et al. investigated a phthalide derivative, levistilide A, to expand functional human umbilical cord blood stem cells *ex vivo*. Following a preliminary screening of natural compounds, levistilide A was selected for further testing. The experimental results showed levistilide A increased the numbers of stem cells and enhanced their colony formation ability. Furthermore, treatment with levistilide A improved rapid engraftment of stem cells with multilineage differentiation. These effects might be related to the reduction of reactive oxygen species (ROS) levels. In this respect other antioxidants should be compared to discriminate between general, ROS-related, protection and potential specific effects of levistilide A. Zhu et al. reported on potential anticancer effects of α-mangostin, a molecule extracted from the hull of the mangosteen. In several *in vitro* assays in different cancer cell lines, α-mangostin increased apoptotic mechanisms, including PARP cleavage, BAX induction, and blockade of AKT signaling, this latter effect was attributed to an α-mangostin-induced degradation of retinoid X receptor. Finally, α-mangostin seemed also active in some *in vitro* assays of migration and invasion. Because most of these effects occur at relatively high (supra micromolar) α-mangostin concentrations, these data deserve further refinement, e. g. lead optimization, to generate a molecular species further testable in *in vivo* settings. Wang et al. attempted to elucidate the mechanisms underlying the antiplatelet effect of berberine and its metabolite berberrubine. Docking and *in vitro* experiments suggested that these compounds inhibit the Rasa3-Rap1 pathway, which is critical for ADP-induced integrin activation (Stefanini et al., 2015). Furthermore, the berberine effect depended on PI3K activation. The antiplatelet effect was confirmed in an *in vivo* thrombosis model (carrageenan-induced thrombosis in mouse tail). The antithrombotic effects of such natural compounds are intriguing, because they point to novel, potentially exploitable, molecular and cellular mechanisms. However, again, additional studies are warranted to achieve, potency, specificity, and selectivity, sufficient for further pharmaceutical development.

Besides the pharmacological characterization of natural compounds, a leading domain in our specialty is Neuropharmacology/Neuroscience, both in terms of discovering novel targetable processes, as well as in terms of studying drug mechanisms, through repurposing and/or pharmacological profiling of new chemical entities. In the Neuropharmacology context, two submissions deal with amyloid-β (Aβ)-related neuroinflammation, two others with potential therapeutic approaches for neuropathic pain. Caruso et al. attempted to elucidate the neuroprotective mechanism of second-generation antidepressants against Aβ-induced neurotoxicity. They report that chronic treatment with vortioxetine or fluoxetine prevented oxidative stress in the hippocampus of Aβ-injected mice and decreased a number of inflammatory markers, including inducible nitric oxide synthase and NADPH oxidase 2, while increased glutathione peroxidase 1, an antioxidant enzyme. Neuroprotection by vortioxetine and fluoxetine was also confirmed *in vitro*, in neuronal cultures challenged with Aβ oligomers. These drugs are commonly used to treat depression associated with Alzheimer’s disease; the present data suggest that they may also exert neuroprotection, which remains to be assessed in clinical settings. The other report by Morgese et al. is also somehow related to Alzheimer’s disease and depression because it attempts to determine whether depression and the associated increase in pain perception occur in an animal model of Aβ-induced neurotoxicity. Intracerebroventricularly Aβ-injected rats displayed memory impairment associated with increased pain susceptibility to mechanical (but not to thermal) stimuli. Many biomarkers were found correlated with the above functional alterations, including increased glutamate, kynurenine, and dopamine and reduced serotonin in the hypothalamus, Cystatin-C, increased, serotonin and melatonin decreased in plasma, with urinary levels paralleling plasma levels. This animal study opens to the possibility of using these biomarkers as an approach for precision medicine.

Kappa opioid agonist have therapeutic potential to treat pain and itch but induce central side effects, such as sedation and dysphoria. Peripherally-restricted kappa-opioid agonists are developed to minimize these adverse effects, because of their scarce penetration in CNS. Wang et al. analyzed the effects of HSK21542, a peripherally-restricted kappa-opioid receptor agonist, in mouse models. HSK21542 showed a brain/plasma concentration ratio of 0.001 and significantly inhibited pain-and hitch-related behaviors, without significant central effects (locomotor activity, respiratory depression). Therefore, this compound seems a promising candidate for treating pain and pruritus and confirms the value of peripherally-restricted kappa-opioid agonists. The other study, by Fidilio et al. tests the hypothesis that reduced levels of transforming growth factor-β1 (TGF-β1) might be involved in aberrant pain processing. By using LP2, a dual-target mu and delta-opioid receptor agonist in the rat chronic constriction injury model, they found a decreased TGF-β1 and TGF-β type II receptor in the spinal cord and rescue of TGF-β1 and TGF-β type II receptor levels in LP2-treated animals. These data suggest that the rescue of TGF-β1 signalling in spinal microglia might be therapeutically relevant and that this might be achieved with the dual opioid receptor targeting approach.

Other reports in this RT deal with mechanisms and targets in cancer, diabetes, chronic inflammation. Lee et al. examined the role of B lymphocyte-induced maturation protein-1 (Blimp-1) in squamous cell carcinoma, in cell lines. They found that a number of stimuli increasing EGF signaling upregulate Blimp-1, while the EGFR inhibitor gefitinib blocks Blimp-1. Moreover, Blimp-1 silencing enhanced cell migration. They conclude that the function of Blimp-1 as a negative regulator of cell migration provides a new therapeutic target in squamous cell carcinoma. Zhang et al. report on the protective effects of polyethylene glycol loxenatide, a long-acting glucagon-like peptide-1 analog, in db/db diabetic mice. They found that, in addition to the known, insulin-related, effect, loxenatide inhibited oxidative stress and decreased pro-inflammatory markers (TNF-α, IL-6, and MCP-1), while increasing anti-inflammatory IL-10. Rong et al. report that photodynamic therapy with a photosensitizer is beneficial for 2,4,6-trinitrobenzene sulfonic acid-induced ulcerative colitis in rats. This treatment seems to act via inhibition of Amine oxidase copper-containing 1 and is accompanied by a decrease of several inflammatory markers.

Finally, two reviews examine the pharmacological potential of some chemical classes of compounds as antimicrobial treatments. Brishty et al. summarize the literature on benzimidazole derivatives, Long et al. the literature on fusidic acid derivatives. Besides the structure-activity information, these reviews also point to potential uses of these derivatives, even in contexts distinct from infectious diseases, such as chronic inflammatory diseases and cancer.
